# Diagnosis between Chronic Hepatitis and Phthisis Pulmonalis

**Published:** 1817-07

**Authors:** 


*For the London Medical and Physical Journal.*

### *Diagnosis between Chronic Hepatitis and Phthisis Pulmonalis; by Dr.* KINGLAKE.

THE origin and extension of diseases are subjects of deep interest and of difficult research. The obscurity attaching to morbid action when the object is to investigate its precise nature and character, will be readily admitted by those who have been most occupied in such inquiries. To mistake one description of complaint for another, is an error into which the most experienced might occasionally fall, and from which the most acute intelligence cannot be wholly exempt. Perhaps no description of medical study is of more importance than that which is employed in correctly discriminating between forms of diseases that have much resemblance to each other, but which, in reality, are essentially different in their nature, in their tendency, and in the mode of treatment that might be necessary for their respective relief and cure.

Several instances have occurred in my practice in which the utmost caution was requisite to distinguish between Chronic Hepatitis and Phthisis Pulmonalis. The former disease, like the latter, has made slow and insidious approaches until it has developed its apparent character as much in symptoms of pulmonic as in those of hepatic affection. The early stage of the hepatic disease is apt to be overlooked and forgotten, whilst its more advanced course is shewn in sympathetic affection of the lungs; and then, and often not till then, it becomes an object of medical treatment. In this unperceived and unsuspected way has chronic hepatitis extended its morbid influence to the thoracic cavity, and has there induced cough, oppression, pain, and expectoration, that have collectively led to a conclusion that the disease was originally and essentially pulmonic.

It has occurred to me to observe four most striking examples of this mis-apprehension, inducing a mode of treatment held to be appropriate for phthisis pulmonalis, without regard having been had to the hepatic disease, and to the biliary and other derangements that had arisen from it. In all these instances, the most hopeless state of phthisis pulmonalis had been imagined, and the usual expectation held as to the inevitable event that awaited the irremediable nature of the affection.

The

The several patients who were the subjects of these cases had been afflicted during many months, and were extremely enfeebled and emaciated. General irritation of the hectic kind, urgent cough, and in two of the instances bloody expectoration, night sweats, &c. prevailed. These patients either had not been bled at all at the time when they came under my care, or so inconsiderably, as in no measure to have reached the exigencies of their respective cases. The action of the pulse, in each of them, was hard, small, and frequent; a sense of weight and uneasiness was referred to the hepatic region; the digestive organs were disordered; and the intestines were generally so obstinately constipated as to require the constant use of laxative medicine. In the persuasion that the mischief originated from either an inflamed, obstructed, or congestive state of the liver, and that the morbid excitement on the chest was a sympathetic extension of ailment only, the treatment that was instituted was directed exclusively to the relief of that organ. Small and repeated bleedings, at intervals of two or three days, during several weeks, with frequent doses of calomel and rhubarb, speedily relieved the symptoms, and afforded the most hopeful prospect of eventual success. This expectation was happily realised; and the several patients are now in health, after a lapse of upwards of three years. More signal and complete success could not attend any curative means, under any circumstances whatever; and the decided and unequivocal nature of the benefit evinces that similar results would, in general, be likely to follow the practice, whenever it be fairly and sufficiently adopted.

It cannot be doubted that a less efficient treatment would have been unavailing for subduing the morbidly increased action of the sanguiferous system.

If the suspicion of original and untractable phthysis pulmonalis had determined the mode of treatment, it would have consisted of palliative expedients rather than of any plan of relief that could have been at all adequate to the demands of the cases; and the attempt would, necessarily, have proved abortive. The fact of each of the cases having been regarded by competent practitioners as phthysis pulmonalis, shews most strongly that the diagnosis of chronic hepatitis and phthysis pulmonalis is ambiguous and difficult. Indeed, where the symptoms common to both affections present under appearances the most imposing and deceptious, it is not easy to avoid being misled. It happens, however, fortunately for the practical advantage of the patient, that the depleting treatment, which would appear to be indis-

 pensably pensably necessary in chronic hepatitis, is as likely to alleviate and remedy the distress of phthysis pulmonalis as any mode of relief that could be pursued; so that it is not probable that any injury would be occasioned in either case by a direct and effectual diminution of vascular distention and excitement. It will be soon found that bleeding, and the use of mercurial medicine in alterative and cathartic doses, will so far relieve the pulmonic symptoms as to leave no doubt that they were the effects of secondary and not of primary affection; and, when this important fact is ascertained, the prospect of cure becomes promising, and will, it may be affirmed, under the treatment proposed, be generally attainable. The destructive career of phthysis pulmonalis is unhappily not so much under controul as that of chronic hepatitis; and it may be justly presumed that the instances on record of the former malady having been arrested in its progress and finally overcome, are examples of the latter disease mistaken for the former, by the resemblance that subsists between these two morbid states.

The diseases of sympathy are as extensive as kindred structure and the associative laws of vital action can render them; and it is of the utmost practical importance correctly to distinguish the affection which is original from that which is sympathetic. Sympathetic diseases are, for the most part, held in existence by the original ailment of which they are the extension; and, as effects, they will generally be found to subside with the removal of the cause. Sometimes the injury inflicted by protracted sympathetic disease becomes a disease of itself independently of the origin from which it proceeds; but this can only happen when a long continuance of the morbid sympathy has either acquired an unyielding habit, or the parts in which it is stationed have undergone change of structure or disorganization. This may be, and often, no doubt, actually is, the case in sympathetic phthysis pulmonalis from hepatic disease, and indicates the urgent necessity of being early and incessant in endeavouring to remove the primary affection, lest that which is secondary should become independent of its cause, and prove inevitably destructive.

*Taunton; May* 10, 1817.

